# Salicylic Acid as an Anaphrodisiac

**Published:** 1878-10

**Authors:** 


Salicylic Acid as an Anaphrodisiac.—A curious fact that may have direct and important bearing on the therapeutics of the sexual organs was brought to light by Dr. C. T. Jewett not long ago, and has since been verified in the case of a veterinary surgeon of Twenty-second street

New York. The doctor found that a patient who had been under his treatment for rheumatism for some time, began to complain that he was no longer able to obtain an erection of the penis. He had been upon fifteen-grain doses of salicylate of soda five times a day for some time. The doctor discontinued the salicylate and put the patient upon damiana, when his sexual power began gradually to return.

The veterinary surgeon put himself upon pretty large doses of salicylic acid for rheumatism, and after a time was surprised at his inability to erect the penis. Although he at once stopped the use of the medicine, he was three months in regaining his sexual power. More extended observations upon the subject are needed.—*American Medical Bi-Monthly.*

